# Comparative Analysis of Mitochondria Surrounding the Intercalated Discs in Heart Diseases—An Ultrastructural Pilot Study

**DOI:** 10.3390/ijms25147644

**Published:** 2024-07-12

**Authors:** Rebecca Schönmehl, Daniel H. Mendelsohn, Lina Winter, Steffen Pabel, Tanja Niedermair, Katja Evert, Wing-Hoi Cheung, Ronald Man Yeung Wong, Volker H. Schmitt, Karsten Keller, Friedrich Barsch, Alexander Dietl, Jan F. Gummert, René Schramm, Samuel Sossalla, Christoph Brochhausen

**Affiliations:** 1 Institute of Pathology, Medical Faculty Mannheim, Heidelberg University, 68167 Mannheim, Germany; 2 Department of Internal Medicine II, University Medical Center Regensburg, 93053 Regensburg, Germany; 3 Institute of Pathology, University of Regensburg, 93053 Regensburg, Germanykatja.evert@klinik.uni-regensburg.de (K.E.); 4 Central Biobank Regensburg, University and University Hospital Regensburg, 93053 Regensburg, Germany; 5 Department of Orthopedics and Traumatology, The Chinese University of Hong Kong, Hong Kong SAR, China; 6 Department of Cardiology, University Medical Centre, Johannes Gutenberg University of Mainz, 55131 Mainz, Germanykarsten.keller@unimedizin-mainz.de (K.K.); 7 German Center for Cardiovascular Research (DZHK), Partner Site Rhine Main, 55131 Mainz, Germany; 8 Center for Thrombosis and Hemostasis (CTH), University Medical Center Mainz, Johannes Gutenberg-University Mainz, 55131 Mainz, Germany; 9 Department of Sports Medicine, Medical Clinic VII, University Hospital Heidelberg, 69120 Heidelberg, Germany; 10 Medical Center, Faculty of Medicine, Institute for Exercise and Occupational Medicine, University of Freiburg, 79106 Freiburg, Germany; 11 Department of Internal Medicine II, University Hospital Regensburg, 93053 Regensburg, Germany; alexander.dietl@ukr.de; 12 Clinic for Thoracic and Cardiovascular Surgery, Heart and Diabetes Center North Rhine-Westphalia, Ruhr University Bochum, 32545 Bad Oeynhausen, Germany; 13 Departments of Cardiology at Kerckhoff Heart and Lung Center, Bad Nauheim and University of Giessen, 61231 Bad Nauheim, Germany

**Keywords:** mitochondria, electron microscopy, ultrastructure, cardiomyopathy, atrial fibrillation, intercalated disc

## Abstract

Background: Mitochondria play a crucial role in adapting to fluctuating energy demands, particularly in various heart diseases. This study investigates mitochondrial morphology near intercalated discs in left ventricular (LV) heart tissues, comparing samples from patients with sinus rhythm (SR), atrial fibrillation (AF), dilated cardiomyopathy (DCM), and ischemic cardiomyopathy (ICM). Methods: Transmission electron microscopy was used to analyze mitochondria within 0–3.5 μm and 3.5–7 μm of intercalated discs in 9 SR, 10 AF, 9 DCM, and 8 ICM patient samples. Parameters included mean size in µm^2^ and elongation, count, percental mitochondrial area in the measuring frame, and a conglomeration score. Results: AF patients exhibited higher counts of small mitochondria in the LV myocardium, resembling SR. DCM and ICM groups had fewer, larger, and often hydropic mitochondria. Accumulation rates and percental mitochondrial area were similar across groups. Significant positive correlations existed between other defects/size and hydropic mitochondria and between count/area and conglomeration score, while negative correlations between count and size/other defects and between hydropic mitochondria and count could be seen as well. Conclusion: Mitochondrial parameters in the LV myocardium of AF patients were similar to those of SR patients, while DCM and ICM displayed distinct changes, including a decrease in number, an increase in size, and compromised mitochondrial morphology. Further research is needed to fully elucidate the pathophysiological role of mitochondrial morphology in different heart diseases, providing deeper insights into potential therapeutic targets and interventions.

## 1. Introduction

Cardiac diseases represent a substantial socio-economic burden, accounting for one in six male and one in ten female deaths [1]. They encompass various conditions, such as coronary artery disease (CAD), (peri-)myocarditis, arrythmias, cardiomyopathies, and valvular heart disease. The increasing incidence of heart diseases underscores the importance of gaining a detailed understanding of the precise underlying pathomechanisms to provide efficient therapies. Notably, recent research has increasingly focused on the role of mitochondria in cardiac diseases [2,3,4]. Due to the dependence of the myocardium on oxidative metabolism, healthy mitochondria are essential to the physiological function of the heart without impairment [5].

In the pathogenesis and development of heart diseases, keeping up with the fluctuating energy demand is a crucial task [6]. Moreover, mitochondrial functions include calcium homeostasis, cell differentiation, and cell death and, thus, play an even greater physiological role than mere energy supply. Furthermore, mitochondrial dynamics, representing the continuous fission and fusion of mitochondria as well as the microtubule-associated transport of mitochondria within the cell, has gained large attention and plays a relevant role in various disorders [7]. Whilst an increasing body of evidence exists regarding the role of mitochondrial dysfunction in heart failure and ischemic cardiomyopathy, including ischemia-reperfusion injury [8], the potential involvement of mitochondrial abnormalities in the pathogenesis of arrythmias is still insufficiently understood. As for the large potential of mitochondria-targeted therapies, it is of utmost importance to reveal the underlying pathophysiology of cardiac mitochondria. 

Interestingly, various cardiac diseases differ in the mitochondrial number, distribution within the cardiomyocyte, and their morphology, making the ultrastructure an interesting aspect to discover. For instance, in patients with tachycardia-induced myopathy (TCM), several studies have identified abnormalities in both mitochondrial distribution and morphology. In addition, the mitochondrial network was consistently observed to shift from the sarcomeres toward the intercalated discs [2,3].

In a comparison of the ultrastructure between normal sinus rhythm (SR) and atrial fibrillation (AF) in patients with rheumatic heart disease, Sharma et al. discovered that AF was linked to an increased number of mitochondria in the left atrial appendage. These mitochondria formed aggregates resembling islands and exhibited variations in size and shape [9]. The potential role of AF for left ventricular (LV) dysfunction is particularly important because clinical studies showed improvement in cardiac function and cardiovascular mortality after AF therapy [10,11].

Examining patients with TCM, Mueller et al. identified a disrupted cardiomyocyte’s mitochondrial distribution pattern with enrichment of mitochondria close to the intercalated discs. Furthermore, electron microscopy revealed a large variation in mitochondrial size and architecture [2]. This finding was confirmed by a study by Paulus et al., who also demonstrated a disturbed mitochondrial distribution and morphology in TCM. 

In the context of the aforementioned intricacies in mitochondrial involvement across diverse cardiac conditions, the present study endeavors to shed light on the ultrastructural nuances of LV mitochondria adjacent to intercalated discs. Using transmission electron microscopy, our focus is to compare mitochondrial populations in patients diagnosed with ischemic cardiomyopathy (ICM) and dilated cardiomyopathy (DCM)—where mitochondrial dysfunction has been extensively studied [12,13]—with those experiencing AF, the most prevalent tachyarrhythmia in modern society [14], and with sinus rhythm (SR).

By our comparative approach, we aim to contribute valuable insights into the specific mitochondrial alterations in the LV associated with distinct cardiac pathologies, paving the way for a deeper understanding and potentially informing novel therapeutic strategies for these prevalent and impactful cardiac conditions.

## 2. Results

Overall, intercalated discs in the left ventricle of 36 patients were examined. The SR group consisted of nine (25.0%) patients with a mean age of 68.44 years (22.22% female, 77.78% male), whereas the AF group included ten (27.8%) patients with a mean age of 70.20 years (0% female, 100% male). The DCM group included nine patients (25.0%) with a mean age of 56.33 years (22.22% female, 77.78% male), and eight (22.2%) patients made up the ICM group with a mean age of 50.25 years (25% female, 75% male) (Table 1).

### 2.1. Distribution of Mitochondria Adjacent (0–3.5 μm Distance) and Proximate (3.5–7 μm Distance) to the Intercalated Discs

Per patient, an area of 500 µm^2^ was evaluated, including 250 µm^2^ adjacent (0–3.5 μm distance) and 250 µm^2^ proximate (3.5–7 μm distance) to the intercalated discs. In the area adjacent (0–3.5 μm distance) to the intercalated discs, a mean percental mitochondrial area of 18.38% for SR, 19.11% for AF, 19.23% for DCM, and 23.33% for ICM was found in the measuring frame (250 µm^2^ per patient). However, mitochondria took up more space in the proximate region (3.5–7 μm distance), leading to a percental mitochondrial area of 28.49% for SR, 29.49% for AF, 29.82% for DCM, and 28.73% for ICM. A significant increase in the percental mitochondrial area in the measuring frame was detected in the groups SR (*p* = 0.0057), AF (*p* = 0.0107), and DCM (*p* = 0.0055). 

A lower average number of mitochondria could be found in the adjacent measuring frames, ranging from 203.6 for DCM to 244.4 for ICM to 261.0 for SR and to 326.7 for AF. In the proximate region, the mean mitochondria count was 297.2 for DCM, 284.5 for ICM, 391.2 for SR, and 490.6 for AF. Although all groups showed a higher number of mitochondria here, this increase was only significant for the SR group (*p* = 0.0209). 

The difference in average mitochondria size in adjacent compared to proximate areas to the intercalated disc could be seen as 0.179 µm to 0.1852 µm for SR, 0.1481 µm to 0.1537 μm for AF, 0.2409 µm to 0.2552 µm for DCM, and 0.2425 µm to 0.2539 µm for ICM. Though not significantly different, all groups showed larger mitochondria in the proximate area. 

The shape of mitochondria was also analyzed by measuring their mean elongation. For all groups, this score was very similar in the region adjacent to the intercalated discs (SR 1.814, AF 1.806, DCM 1.906, and ICM 1.839) compared to the proximate region (SR 1.817, AF 1.771, DCM 1.920, and ICM 1.814). 

The accumulation of mitochondria was measured through the assignment of the conglomeration score. This score varied between groups adjacent to the intercalated discs (SR 20.88, AF 35.82, DCM 10.96, and ICM 17.22), as well as in the proximity of them (SR 37.49, AF 46.58, DCM 20.71, and ICM 22.37). Similar to the increased size, the higher amount of conglomeration in the proximate area, though seen in all groups, was not significant. 

The average percentage of hydropic mitochondria that could be found in the adjacent and the proximate area was 3.33% to 3.36% in SR, 4.62% to 4.56% in AF, 22.39% to 25.28% in DCM, and 15.82% to 18.35% in ICM. Seeing as they were very similar, no significant difference could be found regarding the distance to the intercalated discs. 

The mean number of otherwise defective mitochondria (i.e., lamellar structures arising from mitochondria, loss of cristae without the loss of matrix density) was analyzed as well. In the adjacent area, otherwise defective mitochondria made up 9.66% for AF, 11.56% for SR, 17.46% for DCM, and 17.58% for ICM of the total number of mitochondria seen. Otherwise defective mitochondria could be seen less frequently in proximity to the intercalated discs and made up 6.47% for AF, 6.56% for SR, 12.43% for DCM, and 13.97% for ICM of the total mitochondria in the measuring frame. This decrease in defects was visible for all groups but not significant.

### 2.2. Differences between Groups Regarding Percental Mitochondrial Area in the Measuring Frame, Count, Size, Elongation, and Conglomeration Score, as Well as Percentage of Hydropic and Otherwise Defective Mitochondria

**Figure 1:**
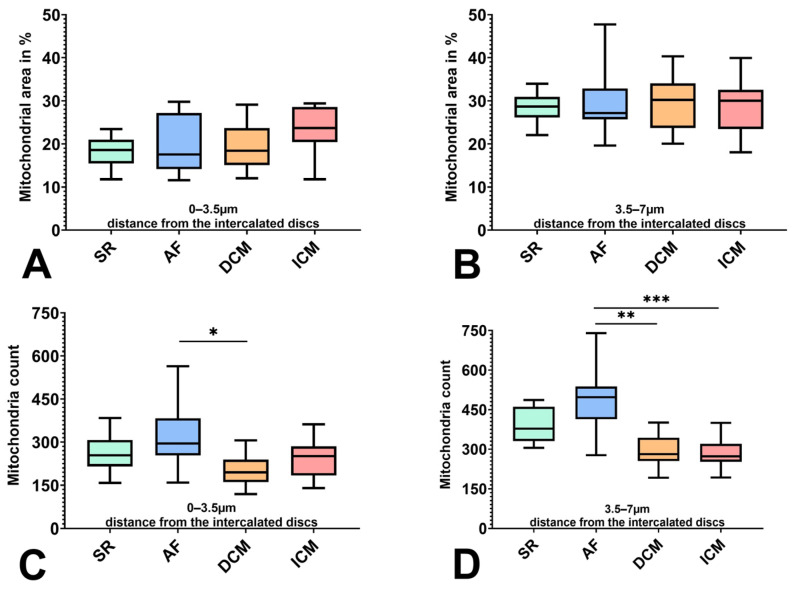
Comparison of the mitochondrial population between patients with SR, AF, DCM, and ICM in 0–3.5 μm (**A**,**C**) and 3.5–7 μm (**B**,**D**) distance from the intercalated discs. (**A**,**B**) Percental mitochondrial area in the measuring frame. (**C**,**D**) Number of mitochondria counted. * indicates statistical significance (* for *p* < 0.05, ** for *p* < 0.01, and *** for *p* < 0.001).

All patient groups showed a similar percental mitochondrial area in the measuring frame (Figure 1A,B). In the area adjacent to the intercalated discs, a mean percental mitochondrial area of 18.38% for SR, 19.11% for AF, 19.23% for DCM, and 23.33% for ICM could be found; in the proximate region, a higher percentage of 28.49% for SR, 29.49% for AF, 29.82% for DCM, and 28.73% for ICM could be seen. In both cases, no significant differences between the groups were made apparent. 

Differences were found in the size and count of the depicted mitochondria. In the adjacent area 0–3.5 µm from the intercalated disc, the AF patients showed a significantly higher count than DCM (*p* = 0.0127) and a significantly smaller size than DCM and ICM mitochondria (*p* = 0.0004 for both). On average, 326.7 mitochondria with a mean size of 0.1481 µm could be seen in the AF group, whereas in the DCM group, 203.6 with a size of 0.2409 µm and in the ICM group, 244.4 with a mean size of 0.2425 µm could be found. 

**Figure 2:**
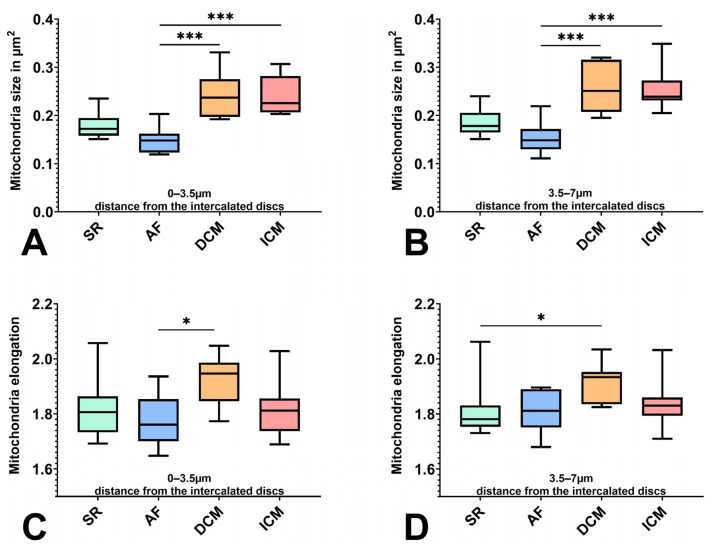
Comparison of the mitochondrial population between patients with SR, AF, DCM, and ICM in 0–3.5 μm (**A**,**C**) and 3.5–7 μm (**B**,**D**) distance from the intercalated discs. (**A**,**B**) Mitochondria size in µm^2^. (**C**,**D**) Mitochondria elongation. * indicates statistical significance (* for *p* < 0.05, and *** for *p* < 0.001).

These results could be seen in the proximate area 3.5–7 μm as well (count: AF vs. DCM *p* = 0.0024; size AF vs. DCM *p* = 0.0006 AF vs. ICM *p* = 0.0004), in addition to a significantly lower number of mitochondria in ICM vs. AF (*p* = 0.0008) (Figure 1C,D and Figure 2A,B). Here, the mean number of mitochondria counted was 391.2 for SR, 490.6 for AF, 297.2 for DCM, and 284.5 for ICM, with mean mitochondria sizes of 0.1852 µm for SR, 0.1537 μm for AF, 0.2552 µm for DCM, and 0.2539 µm for ICM. 

Regarding elongation, the mitochondria of DCM patients generally deviated the most from a round form and showed more heterogeneity. In the region adjacent to the intercalated disc, mitochondria from AF patients were significantly rounder than from the DCM group (*p* = 0.0172); in the proximate region, a similar difference in shape could be observed between SR and DCM mitochondria (*p* = 0.0321) (Figure 2C,D).

**Figure 3:**
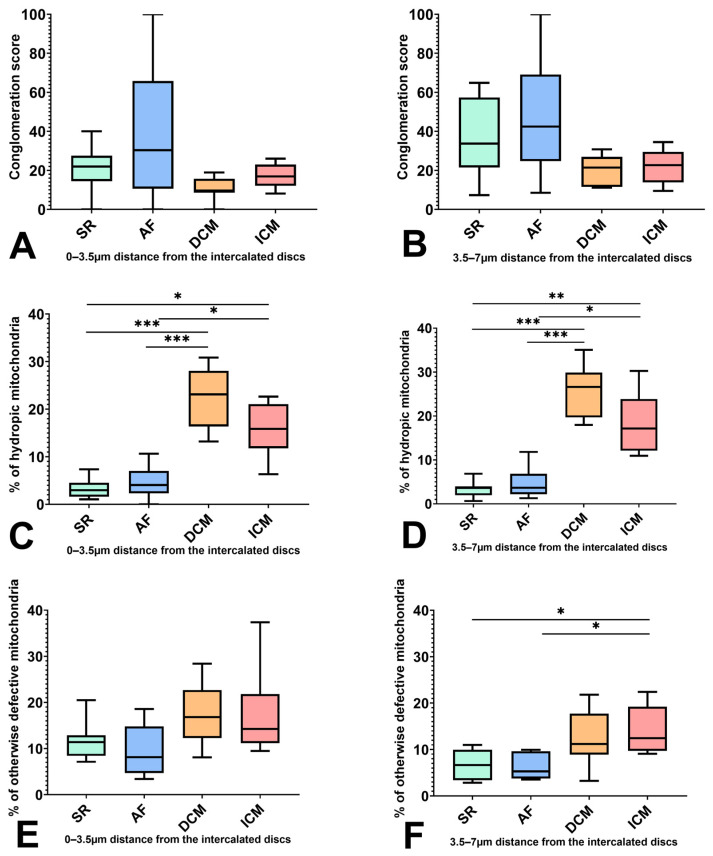
Comparison of the mitochondrial population between patients with SR, AF, DCM, and ICM in 0–3.5 μm (**A**,**C**,**E**) and 3.5–7 μm (**B**,**D**,**F**) distance from the intercalated discs. (**A**,**B**) Conglomeration score. (**C**,**D**) Percentage of hydropic mitochondria. (**E**,**F**) Percentage of otherwise defective mitochondria. * indicates statistical significance (* for *p* < 0.05, ** for *p* < 0.01, and *** for *p* < 0.001).

Although in DCM and ICM mitochondria tended to accumulate less often, no significant difference was found concerning the conglomeration score when compared to their SR and AF counterparts (Figure 3A,B). 

In the adjacent area, a conglomeration score of 20.88 for SR, 35.82 for AF, 10.96 for DCM, and 17.22 for ICM could be calculated. In the proximity of the intercalated disc, accumulation could be measured with conglomeration scores of 37.49 for SR, 46.58 for AF, 20.71 for DCM, and 22.37 for ICM.

A distinct difference in the mitochondrial population was demonstrated in the percentage of hydropic mitochondria. DCM and ICM showed a much higher percentage of hydropic mitochondria at 22.39% and 15.82% than SR and AF patients at 3.33% and 4.62% in the area adjacent to the intercalated discs (0–3.5 µm: SR/AF vs. DCM *p* = 0.0001/0.0005; SR/AF vs. ICM *p* = 0.0116/0.0396). 

This was also seen in the proximate area, where the percentage of hydropic mitochondria made up 25.28% in DCM, 18.35% in ICM, 3.36% in SR, and 4.56% in AF of the seen population (3.5–7 μm: SR/AF vs. DCM *p* = 0.0001/0.0004; SR/AF vs. ICM *p* = 0.0097/0.0245) (Figure 3C,D). 

The percentage of otherwise defective mitochondria was, however, only elevated in ICM patients in the measuring field of 3.5–7 μm (SR/AF vs. ICM *p* = 0.0334/0.0278) (Figure 3E,F). Whereas SR samples showed 6.56% and AF patients showed 6.47% otherwise defective mitochondria, the amount for ICM mitochondria was 13.97%. 

### 2.3. Relationship between the Parameter Percental Mitochondrial Area in the Measuring Frame, Count, Size, Conglomeration Score, and Percentage of Hydropic and Otherwise Defective Mitochondria

Several medium-to-strong relations between parameters could be observed in this study. Positive correlations existed between other defects/size and hydropic mitochondria and between count/area and conglomeration score, while negative correlations between count and size/other defects and between hydropic mitochondria and count could be seen as well.

The number of mitochondria found correlated negatively with the average size (0–3.5 μm: *r*(30) = −0.508, *p* = 0.002; 3.5–7 μm: *r*(30) = −0.747, *p* < 0.001) and with the percentage of otherwise defective mitochondria (0–3.5 μm: *r*(30) = −0.608, *p* = 0.048; 3.5–7 μm: *r*(30) = −0.406, *p* = 0.014). Whereas the count and percentage of otherwise defective mitochondria showed a stronger relation in the proximate area, the relation between the number of mitochondria and size could be observed more clearly in the intercalated disc-adjacent area.

Although it was more evident in the proximate region, the percentage of otherwise defective mitochondria correlated positively with the percentage of hydropic mitochondria in both measuring frames (0–3.5 μm: *r*(30) = 0.484, *p* = 0.003; 3.5–7 μm: *r*(30) = 0.640, *p* < 0.001). A strong relation between the percentage of hydropic mitochondria and their mean size was observed (0–3.5 μm: *r*(30) = 0.685, *p* < 0.001; 3.5–7 μm: *r*(30) = 0.623, *p* < 0.001).

Furthermore, the occurrence of hydropic mitochondria showed a negative correlation to the mitochondria count in both; however, it was more obvious in the proximate region (0–3.5 μm: *r*(30) = −0.430, *p* = 0.009; 3.5–7 μm: *r*(30) = −0.499, *p* = 0.002).

Lastly, a positive relation between conglomeration score and mitochondria counted (0–3.5 μm: *r*(30) = 0.687, *p* < 0.001; 3.5–7 μm: *r*(30) = 0.636, *p* < 0.001) could be seen. A correlation between conglomeration score and percental mitochondrial area in the measuring frame could also be observed (0–3.5 μm: *r*(30) = 0.520, *p* = 0.001; 3.5–7 μm: *r*(30) = 0.566, *p* < 0.001).

## 3. Discussion

With a view to the recent literature, this is the first systematic comparative clinical-pathological study of mitochondrial morphology, size, occupied area, and number of mitochondria adjacent (0–3.5 μm) and proximate (3.5–7 μm) to the intercalated discs in the left ventricular myocardium of patients with atrial fibrillation (AF), ischemic (ICM) and dilated cardiomyopathy (DCM), and sinus rhythm (SR). We identified significant decreases in mitochondrial number along with increases in mitochondrial size in ICM and DCM compared to SR and AF. Furthermore, the results of our study revealed that signs of mitochondrial dysfunction, such as hydropic mitochondria, are significantly more abundant in ICM and DCM than in AF or SR. Additionally, mitochondria from DCM patients were significantly less circular when compared to SR in the proximate region and compared to AF mitochondria in the adjacent region. Although less round, these DCM mitochondria were not necessarily more elongated but more heterogenous in their shape.

The parameters analyzed in the present study correlated with each other in a distinct way. For example, mitochondrial count correlated negatively with size, as well as otherwise defective and hydropic mitochondria. A high mitochondria count was also related to a high conglomeration score. An increased percental mitochondrial area in the measuring frame promoted conglomeration as well. Furthermore, the occurrence of other defects was positively related to the occurrence of hydropic mitochondria. This structural change was seen more often with larger-sized mitochondria as well.

In order to meet the high demands of the heart, cardiomyocytes are largely comprised of contractile myofilament proteins and are reliant on an extensive mitochondrial network to supply these myofilaments with the necessary amount of ATP [15]. Recent studies, however, elucidate the importance of mitochondria for more functions than mere contractility, such as calcium homeostasis, apoptosis, and conductivity. Moreover, it is mentionable that three different mitochondrial populations with distinct properties have been identified in the heart: the perinuclear (PNM), the intermyofibrillar (IFM), and the subsarcolemmal population (SSM) [16]. Due to the immense functional demands of cardiomyocytes coupled with a densely packed cytoplasm, a complex system governing mitochondrial function and distribution within the cell is needed. The underlying molecular mechanisms are, however, insufficiently understood. Although an increasing amount of literature has been identified about the involvement of mitochondrial dysfunction in ischemia-reperfusion injury and heart failure [17,18,19], vastly less is known regarding the role of mitochondria in the pathogenesis of cardiac diseases associated with arrhythmias, such as tachycardiomyopathy (TCM) or AF. It has recently become clear that AF itself exerts detrimental effects on the ventricles, thereby impairing cardiomyocyte function [20]. Clinical studies showed favorable effects of AF ablation on cardiovascular mortality in patients with cardiac disease [11,21]. Our study is the first that evaluates mitochondria in LV tissue from patients with AF. 

In addition to studies illustrating the involvement of mitochondrial dysfunction in the pathogenesis of AF [22], modern research highlights the possible contribution of an altered distribution and morphology of mitochondria to the development of TCM. A study by Paulus et al., for example, elucidated that mitochondria are shifted to the intercalated discs and appear enlarged in TCM-induced rabbit hearts compared to the control group [3]. Interestingly, skinned fibers of the TCM group showed a decrease in the oxidative phosphorylation capacity. Isolated mitochondria, on the other hand, did not exhibit a decrease in oxidative phosphorylation. This indicates that the impaired oxidative phosphorylation of skinned fibers does not result from dysfunctional mitochondria but rather results from a disbalance in the dynamics of the mitochondrial network, including the distribution within the cardiomyocytes.

Similarly, our results showed that mitochondria adjacent (0–3.5 μm) and proximate (3.5–7 μm) to the intercalated disc were significantly (Figure 3) more hydropic and defective in the ICM and DCM groups than in the AF or SR groups, in accordance with a reduced mean ejection fraction in the ICM and DCM groups compared to a normal ejection fraction in the SR and AF groups. Furthermore, these areas consisted of a greater number and smaller mitochondria in the SR and AF groups compared to DCM and ICM, whilst there was no difference in the relative area occupied by mitochondria. This points to a possible shift of the fission–fusion balance towards fission. The results of Paulus et al. demonstrated indifferent fission markers in TCM-induced rabbits, indicating that this process involved a mechanism other than mere fission upregulation but rather an increase in subpopulation-specific fission or alterations in the microtubule-associated distribution of mitochondria. The enrollment of defective or changed microtubule transport in the pathogenesis of AF was postulated by Xiao et al., whose study showed that the application of Taxol, a microtubule stabilizer, prevented AF in an in vitro rabbit heart model [23]. 

Notably, the observed differences in mitochondrial ultrastructure may result from disparities concerning the mean age and gender of the patient groups. The exact impact of gender on mitochondrial function is not yet fully understood, although it has been suggested that estrogen exerts a protective effect on cardiac mitochondria [24]. Ageing is associated with impaired mitochondrial function and altered mitochondrial structure [25,26]. However, the AF group represents the oldest patient group (70.20y) included in this study (Table 1), and yet, mitochondria from the AF group had similar levels of hydropic or otherwise defective mitochondria as the SR group, significantly lower than the DCM or ICM groups (Figure 3). This furthermore outlines the possible role of the distribution of mitochondria within the cardiomyocyte in the pathogenesis of AF. 

Besides mitochondrial ultrastructure, it is also vital to further investigate the involvement of genetic mitochondrial variants in the pathophysiology of cardiac diseases since they may play a pivotal role, as was shown for other diseases [27]. The assessment of antiapoptotic mitochondrial humanins, which are related to mitochondrial damage [28], will be included in future studies.

**Figure 4:**
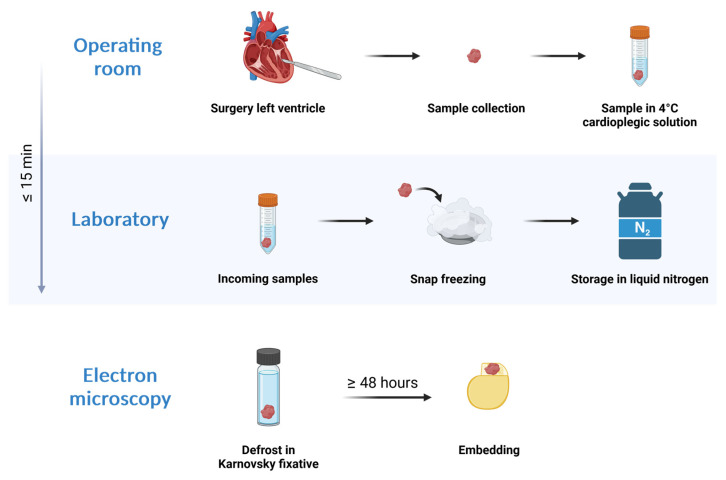
Workflow diagram of sample preparation from acquisition to embedding.

The limitations of this study were the availability of comparable tissue samples and clinical data of the different patient groups, as well as the dependency of the results on the sampling location and the quality of sample preparation (Figure 4, Table 1). Furthermore, the ultrastructural analysis included in this study was conducted on two-dimensional images and thus only represents the state of the mitochondrial population in one level of the heart tissue. To improve our understanding of the role of mitochondria in heart diseases, future studies should perform three-dimensional studies. 

Overall, this pilot study demonstrates alterations in mitochondrial morphology and dynamics across different cardiac diseases. The results also suggest that impairments of mitochondrial function result from more than just direct mitochondrial damage and that the distribution of mitochondria within cardiomyocytes might play a larger role than previously anticipated. In light of promising new treatment strategies targeting mitochondria, it is crucial to further investigate and reveal the exact underlying mitochondrial mechanisms that are involved in cardiomyopathy and arrhythmia. In this context, it is vital to develop new treatments in order to alleviate the socio-economic burden imposed by these diseases.

## 4. Materials and Methods

### 4.1. Sample Acquisition and Patient Characteristics

Ethical approval was granted by the Ethics Committee of the University of Regensburg (reference number: 20-1776-101). Samples of human heart tissue were taken from the left ventricular septum for the groups AF and SR. The samples from AF and SR patients were acquired during open heart surgery from patients with aortic stenosis. LV myectomy was performed for surgical reasons, i.e., because of LV outflow tract obstruction in patients with septal hypertrophy due to aortic stenosis. The SR and AF patients had an overall normal cardiac function and were not different in terms of LV hypertrophy, LV diameter, or severity of aortic stenosis.

For the DCM and ICM groups, samples were taken from the left ventricle, depending on the presence of stents and fibrosis. After removal, all used samples were immediately transferred into a cardioplegic solution (CUSTODIOL^®^, Dr. Franz Köhler Chemie GmbH, Bensheim, Germany) at 4 °C and transported out of the operating room into the laboratory, where they were then snap-frozen in liquid nitrogen. The time from sample acquisition to freezing was typically no longer than 15 min (Figure 4). 

A total of 36 patients were included in this study (SR/AF/DCM/ICM *n* = 9/10/9/8), for which the patient data are shown in Table 1. 

### 4.2. Sample Preparation

For the ultrastructural analysis, the tissue was allowed to thaw in Karnovsky Fixative (an aqueous buffered glutaraldehyde solution) for at least 48 h and was then embedded (post-fixation with osmium tetroxide, dehydration, infiltration with EPON) in the LYNX microscopy tissue processor (Reichert-Jung, Wetzlar, Germany). Semi-thin sections (75 nm) for the selection of relevant areas and ultra-thin sections (80 nm) were cut using the Leica Ultracut S Microtome (Leica-Reichert, Wetzlar, Germany). 

The ultra-thin sections were then contrasted with aqueous 2%-uranyl-acetate and 2%-lead-citrate solutions for 10 min each. For the sample documentation, the 2k × 2k side-mounted camera of an LEO 912AB electron microscope (Zeiss, Oberkochen, Germany) was used with iTEM software version 5.2 (OSIS, Muenster, Germany).

### 4.3. Evaluation

For the evaluation of the ultrastructural images, the software RADIUS version 2.2 (EMSIS, Muenster, Germany) was used. For the selection of the measuring field, frames were drawn at 0–3.5 µm and 3.5–7 μm distance to the intercalated discs. The width of the frames was determined individually by the avoidance of interstitial space and otherwise unusable areas. An area of 500 µm^2^ (250 µm^2^ in 0–3.5 μm, 250 µm^2^ in 3.5–7 μm) consisting of frames from multiple intercalated discs/images was analyzed per patient. In each measuring field, the mitochondria were counted and marked. This evaluation was carried out in a blinded manner.

The parameters we evaluated from the initial annotation were percental mitochondrial area in the measuring frame, mitochondria count, mean size in µm^2^, and the elongation of the mitochondria. Elongation is defined as the squared quotient of the longitudinal and transversal deviation of all pixels belonging to the object along the regression line and takes the minimum value of 1 for a circle.

**Figure 5:**
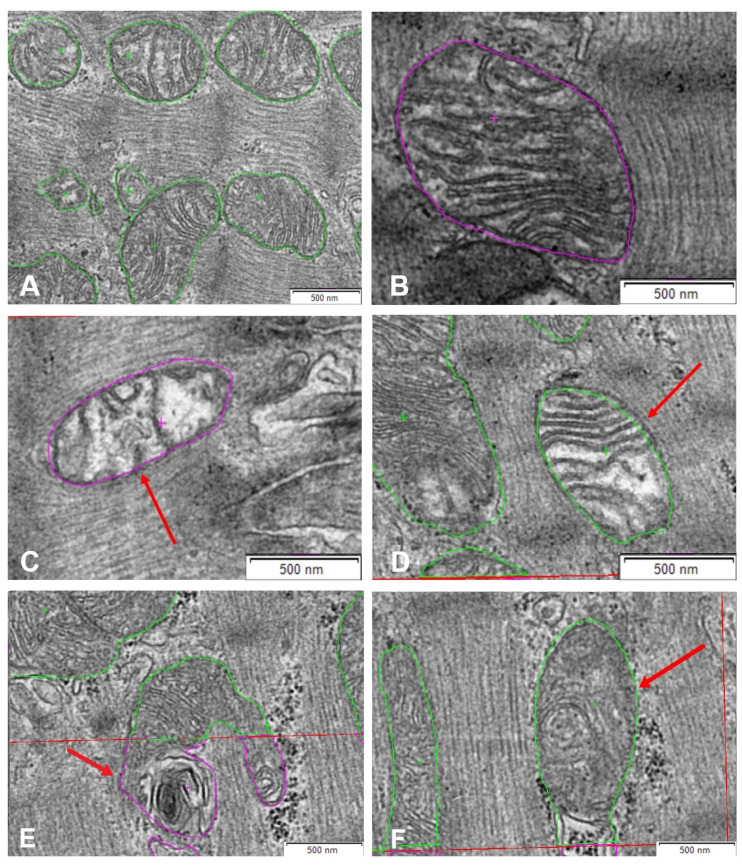
Comparison of mitochondrial ultrastructure. (**A**,**B**) Healthy mitochondria (electron-dense with regular cristae structure). (**C**,**D**) Hydropic mitochondria marked with red arrows (reduced matrix density). (**E**,**F**) Otherwise defective mitochondria marked with red arrows (lamellar structures arising from mitochondria, loss of cristae without the loss of matrix density); pink-labeled mitochondria are adjacent and green-labeled mitochondria are proximate to the intercalated disc.

After that, mitochondrial health was evaluated by counting healthy (Figure 5A,B) hydropic and otherwise defective forms (Figure 5). We defined mitochondria as hydropic by their decreased matrix density according to the literature [29] (Figure 5C,D). In addition, we used the mean grey value of the mitochondria as a way to compare their matrix density. Since illumination times differed between images, we compared borderline cases of hydropic mitochondria to non-defective mitochondria in the same image. All mitochondria categorized as hydropic were at least 5% lighter regarding their mean grey value than regular mitochondria in the same image. 

Mitochondria showing other defects, such as lamellar structures arising from mitochondria [30] or the loss of cristae without the loss of matrix density, were defined as “otherwise defective” (Figure 5E,F). 

**Figure 6:**
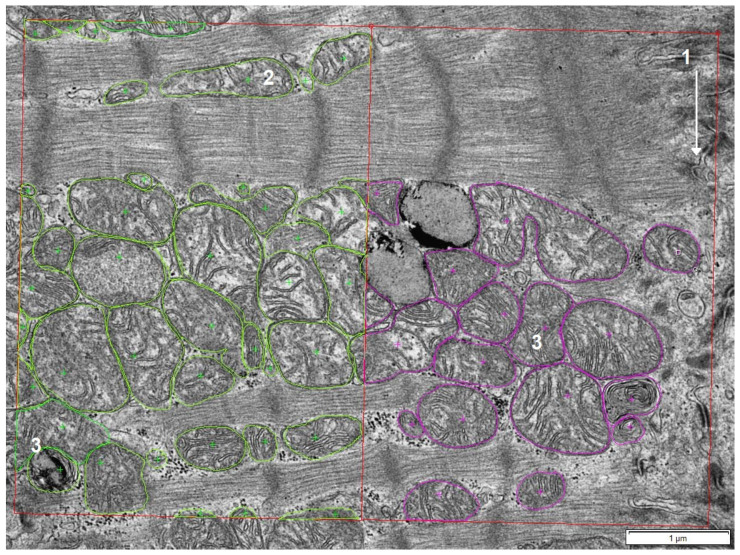
Measuring frames adjacent at 0–3.5 µm and 3.5–7 µm distance to the intercalated disc (1) with regular interfibrillar (2) and conglomerated (3) mitochondria; pink-labeled mitochondria are adjacent and green-labeled mitochondria are proximate to the intercalated disc.

Next, a conglomeration score from 0–100 was assigned to each patient. It was necessary to have measuring frames of varying sizes due to differences in the length of the intercalated disc and other excluding factors, such as interstitial spaces seen in each image. For this reason, the analyzed area of 500 µm^2^ (250 µm^2^ in 0–3.5 µm and 250 µm^2^ in 3.5–7 μm distance to the intercalated disc) consisted of 10–14 frames per patient. If a frame showed a cluster of mitochondria that was no longer situated in between but overgrew the muscle fibers, this frame was defined as conglomerated (Figure 6). Depending on the size of the measuring frame, the area that showed conglomeration was weighted proportionally to the total analyzed area per patient.

### 4.4. Statistical Analysis

The statistical analysis of the data was conducted using GraphPad Prism version 9.5.1 (GraphPad Software, San Diego, CA, USA). A one-way ANOVA Kruskal–Wallis test (alpha = 0.05) followed by a Dunn’s test was performed to identify differences between the analyzed heart damage regarding percental mitochondrial area in the measuring field, size, count, conglomeration, swelling, and other defects. 

A Spearman correlation (alpha = 0.05, two-tailed *p*-value) was used to investigate the relation between the above-mentioned parameters as well as complementary patient data. All *p*-values ≤ 0.05 were considered statistically significant. 

## 5. Conclusions

In conclusion, this systematic comparative clinical–pathological pilot study has provided novel insights into the mitochondrial morphology, size, occupied area, and number of mitochondria in cardiomyocytes from patients with atrial fibrillation (AF), ischemic cardiomyopathy (ICM), dilated cardiomyopathy (DCM), and sinus rhythm (SR) in regions adjacent (0–3.5 μm) and proximate (3.5–7 μm) to the intercalated discs in left ventricular heart tissue. Significant alterations in mitochondrial parameters were observed, with increased mitochondrial number and decreased size in AF and SR compared to ICM and DCM. Additionally, morphological signs of mitochondrial dysfunction were more pronounced in ICM and DCM. 

Despite advancements in understanding mitochondrial dysfunction in ischemia-reperfusion injury and heart failure, less is known about its role in cardiac diseases affecting conductivity, such as tachycardiomyopathy (TCM) and AF. Our findings, in line with recent research, suggest that altered mitochondrial distribution may be a contributing factor to the development of arrhythmias.

Since recent studies showed that AF itself has detrimental effects on the ventricles and is thereby impairing cardiomyocyte function, it was a novel approach of our study to especially compare left ventricular heart tissue.

In summary, our study highlights the intricate relationship between mitochondrial alterations and cardiac diseases. It underscores that impairments in mitochondrial function extend beyond direct damage and may involve the distribution of mitochondria within cardiomyocytes. As new treatment strategies targeting mitochondria emerge, further investigation into the underlying mitochondrial mechanisms is essential. Understanding these mechanisms will be instrumental in developing effective treatments to alleviate the socio-economic burden imposed by cardiomyopathy and arrhythmias.

## Figures and Tables

**Table 1:** Patient characteristics: sex distribution, mean age, and heart rhythm.

Group	Sample Size	Female		Male		Age	SR		VHF		Pacemaker Rhythm	
		n	%	n	%		n	%	n	%	n	%
SR	9	2	22.22	7	77.78	68.44	9	100.00	0	0.00	0	0.00
AF	10	0	0.00	10	100.00	70.20	0	0.00	10	100.00	0	0.00
DCM	9	2	22.22	7	77.78	56.33	5	55.56	3	33.33	1	11.11
ICM	8	2	25.00	6	75.00	50.25	5	62.50	3	37.50	0	0.00
Overall	36	6	16.67	30	83.33	61.86	19	52.78	16	44.44	1	2.78

## Data Availability

Data are contained within the article.
